# Prevalence and clinical correlation of hepatitis E virus antibody in the patients’ serum samples from a tertiary care hospital in Thailand during 2015–2018

**DOI:** 10.1186/s12985-021-01616-x

**Published:** 2021-07-12

**Authors:** Atiporn Boonyai, Anchalee Thongput, Thidarat Sisaeng, Parisut Phumchan, Navin Horthongkham, Wannee Kantakamalakul, Chutikarn Chaimayo

**Affiliations:** Department of Microbiology, Faculty of Medicine Siriraj Hospital, Mahidol University, Bangkok, Thailand

**Keywords:** Hepatitis E, HEV, Seroprevalence, Incidence, Transplant, Pregnancy, Thailand

## Abstract

**Background:**

Prevalence and incidence of hepatitis caused by HEV infection are usually higher in developing countries. This study demonstrated the HEV seroprevalence and incidence of HEV infection in patients with clinical hepatitis in a tertiary hospital in Thailand.

**Methods:**

A laboratory-based cross-sectional study was conducted using 1106 serum samples from patients suspected of HEV infection sent to the Serology laboratory, Siriraj Hospital, for detecting HEV antibodies during 2015–2018. Prevalence of anti-HEV IgG and IgM antibodies in general patients, including organ transplant recipients and pregnant women in a hospital setting, were determined using indirect enzyme-linked immunosorbent assay (ELISA) kits. Comparison of laboratory data between groups with different HEV serological statuses was performed.

**Results:**

HEV IgG antibodies were detected in 40.82% of 904 serum samples, while HEV IgM antibodies were detected in 11.75% of 1081 serum samples. Similar IgG and IgM antibody detection rates were found in pregnant women. Interestingly, anti-HEV IgM antibodies were detected in 38.5% of patients who underwent organ transplantation. Patients who tested positive for anti-HEV IgM antibodies had higher alanine aminotransferase levels than those who had not. In contrast, patients who tested positive for anti-HEV IgG had more elevated levels of total bilirubin than those who tested negative.

**Conclusions:**

HEV seroprevalence and incidence in patients with clinical hepatitis were relatively high in the Thai population, including the pregnancy and organ transplant subgroups. The results potentially benefit the clinicians in decision-making to investigate HEV antibodies and facilitating proper management for patients.

## Background

Viral hepatitis is a condition characterized by inflammation and necrosis of the liver caused by hepatitis viruses, including hepatitis A virus (HAV), hepatitis B virus (HBV), hepatitis C virus (HCV), hepatitis D virus (HDV), and hepatitis E virus (HEV). HEV is an under-recognized emerging virus, which is transmitted via consuming contaminated food or water [1]. The incidence of acute hepatitis caused by HEV infection is higher in developing countries than in developed countries [2]. Infections in young children are usually asymptomatic, while infections in pregnant women can lead to detrimental fetal and maternal outcomes. Risk factors associated with symptomatic HEV infection and more severe clinical outcomes are immunocompromised statuses, including organ transplantation, liver cirrhosis, very old age (> 80 years), and pregnancy [3–8].

HEV seroprevalence in Thai individuals, determined by positive anti-HEV IgG antibodies, was previously reported by Poovorawan et al., 1996 as 9–22%. The rate of acute HEV infection, defined as positive anti-HEV IgM antibodies, was around 7% in this study [9]. A study in young Thai men by Gonwong et al*.* [10] revealed a seroprevalence of HEV of 14%. Recently, Jupattanasin et al*.* [11] reported a 29.7% anti-HEV prevalence in Thai blood donors. The higher prevalence was observed in specific subpopulations, i.e., kidney and liver transplant recipients, ranging from 26 to 56% [12–15].

The Serology laboratory, Department of Microbiology, Faculty of Medicine Siriraj Hospital, has provided the service for anti-HEV IgG and anti-HEV IgM testing since June 2014. Increased requests for HEV serological tests were observed during the following years, suggesting that the seroprevalence of HEV could be different from what has been observed in previous studies. This cross-sectional study investigated the recent HEV seroprevalence and incidence of acute HEV infection in patients at a tertiary hospital in Thailand during 2015–2018, including but not limited to those who were pregnant or underwent organ transplantation. Clinical correlation of patients with different HEV serological status was also observed. Knowing the HEV prevalence and the potential risk factors for severe cases will raise the awareness for disease recognition and HEV burden. The outcome of the study can benefit the clinicians in deciding whether to investigate for HEV antibodies and prompt the laboratory service to prepare for the HEV epidemic.

## Methods

### Ethical statement

This study was approved by the Institutional Review Board of The Faculty of Medicine Siriraj Hospital, Mahidol University (SIRB protocol 720/2561(IRB4); COA: Si 040/2019).

### Study design and sample collection

The study design is a retrospective laboratory-based cross-sectional study, single-center site. A total number of 1,106 clotted blood samples of patients suspected of hepatitis E virus infection was sent to the Serology laboratory, Department of Microbiology, Faculty of Medicine Siriraj Hospital, Mahidol University, Bangkok, Thailand from January 2015 to December 2018 for investigation of HEV antibodies. Blood samples, which were requested by the physicians to test for both anti-HEV IgG and IgM antibodies were included for the clinical and laboratory data analysis. The serial blood samples which obtained from the same patients during the experimental period and revealed the same results were excluded from the study. Sera were separated from the blood samples by centrifugation at 3500 rpm for 15 min.

### Serology test

Serum samples were examined for HEV antibodies using Anti-Hepatitis E virus (HEV) IgG and Anti-Hepatitis E Virus (HEV) IgM ELISAs (EUROIMMUN, Federal Republic of Germany) according to the manufacturer’s instruction. The detection principle was indirect ELISA based on the binding of HEV antibodies (IgG/IgM) in sera to HEV recombinant antigens (genotype 1 and 3). The cut-off value of ≥ 1.1 was regarded as positive, while those value less than 0.8 was regarded as negative. The values between 0.8 and 1.1 were considered borderline.

### Statistical analysis

SPSS Statistics version 18.0 was used for statistical analysis. General information of patients was described using descriptive statistics. Continuous data were presented in median and range. Comparison of laboratory data between groups with different HEV serological status was performed using the Kruskal–Wallis test due to the non-normal distribution. Categorical data were presented in numbers and percentages. Associations between categorical variables were analyzed using the Chi-square test. A *P-value* less than 0.05 is considered statistical significance.

## Results

### Prevalence of anti-HEV IgG in the study population

During 2015–2018, a total of 1,106 serum samples of suspected HEV-infected cases were sent to the Serology laboratory, Siriraj Hospital, for the detection of HEV IgM/IgG antibodies. A total number of 904 blood samples were sent for anti-HEV IgG antibody detection during 2015–2018. There were 138, 202, 276, and 288 serum samples in 2015, 2016, 2017, and 2018, respectively. On the contrary, the detection rate of positive anti-HEV IgG antibody decreased over time. HEV IgG antibody positive rate significantly decreased by time, i.e. 65.94%, 49.01%, 37.68%, and 26.04% in 2015, 2016, 2017 and 2018, respectively (*p* < 0.001). Cumulatively, HEV IgG antibodies were detected in 369 out of 904 (40.82%) serum samples (Table 1).

**Table 1:** Percentages of positive anti-HEV IgG and IgM in patients at Siriraj Hospital during 2015–2018

Year	Positive anti-HEV IgG/Total (%)	Positive anti-HEV IgM/Total (%)
2015	91/138 (65.94)	38/165 (23.03)
2016	99/202 (49.01)	27/230 (11.74)
2017	104/276 (37.68)	34/331 (10.27)
2018	75/288 (26.04)	28/355 (7.89)
Total	369/904 (40.82)	127/1081 (11.75)

Numbers of positive anti-HEV IgG and anti-HEV IgM serum samples/total samples were demonstrated in percentages per year (2015–2018) with a Chi-square test for trend *P-*value < 0.001*

**Fig. 1:**
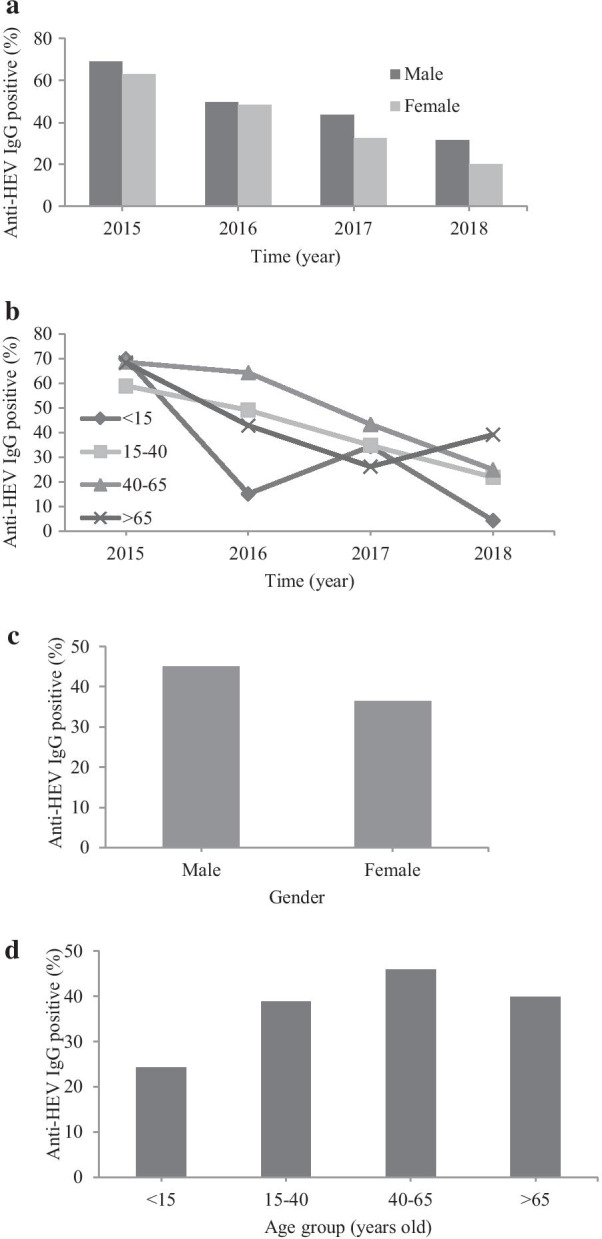
Anti-HEV IgG detection rate in serum samples of patients sent to serology laboratory, Siriraj Hospital during 2015–2018. **a** Percentage of positive anti-HEV IgG serum samples sorted by gender per year, **b** Percentage of anti-HEV IgG positive sorted by age group per year, **c** Cumulative anti-HEV IgG detection rate during 2015–2018 in serum samples sorted by gender, **d** Cumulative anti-HEV IgG detection rate during 2015–2018 in serum samples sorted by age group

We analyzed the sampling year group according to gender and found higher prevalence of anti-HEV IgG in men than women (45.05% versus 36.53%; *p* = 0.009) (Table 2, Fig. 1A, C). Anti-HEV IgG antibodies significantly differed among age groups (*p* < 0.001). The highest positive rate was found in the age group 40–65 years old (45.97%; 194/422), while the lowest positive rate was found in the age group < 15 years old (24.21%; 23/95) (Table 3, Fig. 1B, D).

**Table 2:** Percentages of positive anti-HEV IgG in male and female cases at Siriraj Hospital during 2015–2018

Year	Positive anti-HEV IgG/Total (%)		Chi square test, *P-*value
	Male	Female	
2015	47/68 (69.12)	44/70 (62.86)	0.602, *p* = 0.438
2016	55/111 (49.55)	44/91 (48.35)	0.029, *p* = 0.865
2017	57/131 (43.51)	47/145 (32.41)	3.610, *p* = 0.057
2018	46/145 (31.72)	29/143 (20.28)	4.896, *p* = 0.027*
Total	205/455 (45.05)	164/449 (36.53)	6.806, *p* = 0.009*

Numbers of positive anti-HEV IgG serum samples/total samples in male and female patients were demonstrated in percentages per year (2015–2018). Chi-square test was used to analyze the association between gender and anti-HEV IgG seropositivity. A *P*-value less than 0.05 is considered statistical significance

**Table 3:** Percentages of positive anti-HEV IgG in different age groups at Siriraj Hospital during 2015–2018

Year	Positive anti-HEV IgG/Total (%)				
	Age (years old) < 15	15–40	40–65	> 65	Chi square test, *P-value*
2015	7/10 (70.00)	23/39 (58.97)	48/70 (68.57)	13/19 (68.42)	1.184, *p* = 0.757
2016	5/33 (15.15)	28/57 (49.12)	54/84 (64.29)	12/28 (42.86)	23.406 *p* < 0.001*
2017	10/29 (34.48)	24/69 (34.78)	59/136 (43.38)	11/42 (26.19)	4.617, *p* = 0.202
2018	1/23 (4.35)	14/64(21.88)	33/132 (25.00)	27/69 (39.13)	12.409, *p* = 0.006*
Total	23/95 (24.21)	89/229 (38.86)	194/422 (45.97)	63/158 (39.87)	15.906, *p* = 0.001*

Numbers of positive anti-HEV IgG serum samples/total samples in patients with different age groups were demonstrated in percentages per year (2015–2018). Chi-square test was used to analyze the association between age group and anti-HEV IgG seropositivity. A *P-value* less than 0.05 is considered statistical significance

### Laboratory confirmed acute HEV infection in the study population

Acute HEV infection can be laboratory confirmed by the detection of anti-HEV IgM antibodies. A total number of 1,081 blood samples were sent for anti-HEV IgM antibody detection during 2015–2018. There were 165, 230, 331, and 355 serum samples in the year 2015, 2016, 2017, and 2018, respectively. Similar to that of ani-HEV IgG, number of serum samples sent for detection of anti-HEV IgM increased every year, while the detection rate decreased over time from 23.03%, 11.74%, 10.27%, and 7.79% in 2015, 2016, 2017 and 2018, respectively (*p* < 0.001). Cumulatively, HEV IgM antibodies were detected in 127 out of 1,081 (11.75%) serum samples (Table 1).

**Fig. 2:**
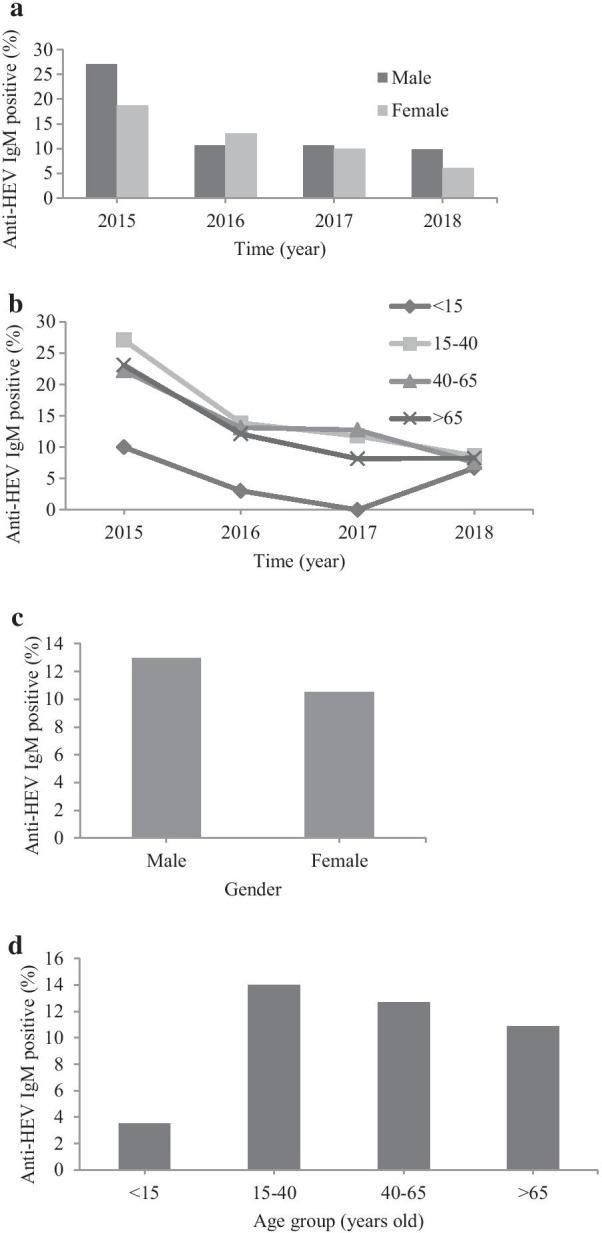
Anti-HEV IgM detection rate in serum samples of patients sent to serology laboratory, Siriraj Hospital during 2015–2018. **a** Percentage of positive anti-HEV IgM serum samples sorted by gender per year, **b** Percentage of anti-HEV IgM positive sorted by age group per year, **c** Cumulative anti-HEV IgM detection rate during 2015–2018 in serum samples sorted by gender, **d** Cumulative anti-HEV IgM detection rate during 2015–2018 in serum samples sorted by age group

We found no significant difference of positive anti-HEV IgM antibody detection rate between men and women each year nor in four-year cumulative duration (12.96% versus 10.54%; *p* = 0.215) (Table 4, Fig. 2A, C). Anti-HEV IgM antibodies significantly differed among age groups (*p* < 0.001). The highest positive rate of HEV IgM antibodies was detected in the age group 15–40 years old (13.98%; 39/279), while the lowest positive rate was found in the age group < 15 years old (3.54%; 4/113) (Table 5, Fig. 2B, D).

**Table 4:** Percentages of positive anti-HEV IgM in male and female cases in Siriraj Hospital during 2015–2018

Year	Positive anti-HEV IgM/Total (%)		Chi square test, *P-value*
	Male	Female	
2015	23/85 (27.06)	15/80 (18.75)	1.605, *p* = 0.205
2016	13/123 (10.57)	14/107 (13.08)	0.349, *p* = 0.554
2017	17/160 (10.63)	17/171 (9.94)	0.042, *p* = 0.838
2018	17/172 (9.88)	11/183 (6.01)	1.830, *p* = 0.176
Total	70/540 (12.96)	57/541 (10.54)	1.535, *p* = 0.215

Numbers of positive anti-HEV IgM serum samples/total samples in male and female patients were demonstrated in percentages per year (2015–2018). Chi-square test was used to analyze the association between gender and anti-HEV IgM seropositivity. A *P-value* less than 0.05 is considered statistical significance

**Table 5:** Percentages of positive anti-HEV IgM in different age groups at Siriraj Hospital during 2015–2018

Year	Positive anti-HEV IgM/Total (%)				Chi square test, *P-value*
	Age (years old) < 15	15–40	40–65	> 65	
2015	1/10 (10.00)	13/48 (27.08)	18/81 (22.22)	6/26 (23.08)	1.433, *p* = 0.698
2016	1/33 (3.03)	9/65 (13.85)	13/99 (13.13)	4/33 (12.12)	2.884, *p* = 0.410
2017	0/40 (0.00)	10/85 (11.76)	20/157 (12.74)	4/49 (8.16)	–
2018	2/30 (6.67)	7/81 (8.64)	12/159 (7.55)	7/85 (8.24)	0.165, *p* = 0.983
Total	4/113 (3.54)	39/279 (13.98)	63/496 (12.70)	21/193 (10.88)	9.257, *p* = 0.026*

Numbers of positive anti-HEV IgM serum samples/total samples in patients with different age groups were demonstrated in percentages per year (2015–2018). Chi-square test was used to analyze the association between age group and anti-HEV IgM seropositivity. A *P-value* less than 0.05 is considered statistical significance

### Clinical and laboratory data of patients classified by the serological status of HEV

Anti-HEV IgM antibody can be detectable 3–4 days after the disease onset and may remain in the serum up to 5–6 months. While anti-HEV IgG antibody usually rises a few days after the presence of anti-HEV IgM and lasts for many years after infection [16]. To classify the patients according to the stage of HEV infection based on their HEV serological status, four subgroups including group I: negative anti-HEV IgG and IgM (G−/M−), group II: positive anti-HEV IgG with negative anti-HEV IgM (G+/M−), group III: negative anti-HEV IgG with positive anti-HEV IgM (G−/M+), and group IV: positive anti-HEV IgG and IgM (G+/M+) were analyzed for their liver function test. Of 1106 serum samples, 879 were sent for the detection of both anti-HEV IgG and IgM antibodies. The results were reported as negative, borderline, and positive for anti-HEV IgG and IgM. The borderline results were excluded for subgroup analysis, resulting in 796 serum samples being analyzed. There were 456 samples (57.29%) in group I (G−/M−), 233 samples (29.27%) in group II (G+/M−), 6 samples (0.75%) in group III (G−/M+), and 101 samples (12.69%) in group IV (G+/M+) (Table 6).

**Table 6:** Laboratory data of patients classified by serological status of HEV during 2015–2018

Laboratory data	Age (years)	AST (U/L)	ALT (U/L)	ALP (U/L)	Total bilirubin (mg/dL)
All cases (n = 796)	49 (0.03–94)	204 (10–7778)	217 (6–7000)	163 (32–3346)	2.9 (0.2–56.7)
Group I	48 (0.08–94)	195 (10–7778)	236 (7–6247)	163 (32–3346)	2.4 (0.2–49.4)
HEV IgG− HEV IgM− (n = 456)					
Group II	50 (0.08–93)	185 (14–7000)	167 (6–7000)	170 (41–1422)	4.3 (0.2–56.7)
HEV IgG+ HEV IgM− (n = 233)					
Group III	48.5 (4–59)	135.5 (30–915)	330 (19–1974)	212.5 (173–348)	1.6 (0.2–9.9)
HEV IgG− HEV IgM+ (n = 6)					
Group IV	50 (0.03–91)	262 (16–3408)	343 (7–3383)	163 (51–1348)	3.9 (0.3–36.7)
HEV IgG+ HEV IgM+ (n = 101)					
*P-*value	0.125	0.224	0.039*	0.573	0.017*

A total number of 796 serum samples, which were sent for detection of both anti-HEV IgG and IgM antibodies, were analyzed according to the HEV serological status. General patient information and laboratory data, including age, AST, ALT, ALP, and total bilirubin, were demonstrated in median and range. Distributions across four HEV serological statuses (Group I: HEV IgG−/IgM−, Group II: HEV IgG+/IgM−, Group III: HEV IgG−/IgM+, and Group IV: HEV IgG+/IgM+) were compared using the Kruskal–Wallis test. A *P-value* less than 0.05 is considered statistical significance

We reviewed the clinical data and laboratory results of patients with different HEV serological statuses. The median levels of aspartate aminotransferase enzyme (AST) and alkaline phosphatase (ALP) did not differ across the subgroups (*P*-value = 0.224 for AST; 0.573 for ALP). Of note, the median levels of alanine aminotransferase enzyme (ALT) and total bilirubin were significantly different across the four HEV serological subgroups. Patients who were tested positive for anti-HEV IgM had higher ALT levels compared to those with negative anti-HEV IgM (*P*-value = 0.039). The most elevated median ALT, which was 343 (7–3383) U/L, was found in patients from group IV (G+/M+), while the lowest, which was 167 (6–7000) U/L, was found in patients from group II (G+/M−). Interestingly, patients who tested positive for anti-HEV IgG had higher total bilirubin levels than those with negative anti-HEV IgG (*P*-value = 0.017). The most elevated median total bilirubin, which was 4.3 (0.2–56.7) U/L, was found in patients from group II (G+/M−), while the lowest, which was 1.6 (0.2–9.9) U/L, was found in patients from group III (G−/M+) (Table 6).

### HEV serological status in the pregnancy group

Pregnant women are among the high-risk HEV infected patients. In this study, women in the pregnancy group were defined as women with pregnancy status at the time of blood collection to investigate anti-HEV antibodies or women whose pregnancy status was terminated within two weeks after having an anti-HEV antibody test. Of 1,106 serum samples sent to the Serology laboratory for anti-HEV antibody test, 17 samples (1.54%) were from the pregnancy group. The median age of this subpopulation was 32 years old (range 17–41). Most pregnant women were in their third trimester (82.4%). Of these 17 pregnant women, eight were presented with hyperbilirubinemia (47.1%), while 13 were presented with transaminitis with elevation of AST or ALT more than 100 U/L (76.5%). Five patients were diagnosed with acute fatty liver in pregnancy and four were diagnosed with viral hepatitis (1 HAV, 1 HBV, and 2 HEV infections). Ectopic pregnancy (n = 1), DFIU (n = 1), threaten preterm labor/preterm labor (n = 3), and low infant birth weights (n = 10) were observed as pregnancy and fetal complications (Table 7).

**Table 7:** Clinical and laboratory data of patients in the pregnancy group during 2015–2018

Characteristics	Results
Number of pregnancy cases	n = 17 (17 out of 1,106; 1.54%)
Age (years)	32 (range 17–41)
Gravidity	G1: n = 9 (52.9%), G2: n = 5 (29.4%), G3: n = 3 (17.6%)
Twin/Triplet pregnancy	n = 3 (17.6%)
Gestational age	First trimester: n = 1 (5.9%), Second trimester: n = 2 (11.8%), Third trimester: n = 14 (82.4%)
Clinical/laboratory presentations	
Hyperbilirubinemia	n = 8 (47.1%)
Elevation of AST or ALT > 100 U/L	n = 13 (76.5%)
Liver function test (All 17 cases)	
AST (U/L)	143 (24–1,576)
ALT (U/L)	242 (19–1,708)
ALP (U/L)	196 (57–500)
TB (mg/dL)	2.04 (0.3–24.3)
Diagnoses	
Recent viral hepatitis	n = 4 (23.5%); HAV (n = 1), HBV (n = 2), HEV (n = 2)
Acute fatty liver in pregnancy	n = 5 (29.4%); one was also diagnosed with syphilis
Pre-eclampsia/HELLP	n = 3 (17.6%)
Others	ATV/r induced hyperbilirubinemia (n = 1), chronic HT with UGIB (n = 1), acute/post-arrest hepatitis (n = 2), pregnancy with DFIU (n = 1)
Pregnancy and fetal complications	
Ectopic pregnancy/DFIU	n = 2 (11.8%)
Threaten preterm labor/preterm labor	n = 3 (17.6%); from maternal HAV infection (n = 1), maternal HBV infection (n = 1)
Low infant birth weight (< 2,500 g)	n = 10 (58.9%); from maternal HAV infection (n = 1), maternal HBV infection (n = 1), maternal HEV infection (n = 1)
Timing of anti-HEV antibody test	Pre-partum: n = 7 (41.2%), Peri/Post-partum: n = 10 (58.9%)
Results of anti-HEV antibody test	
Positive anti-HEV IgG antibody	n = 7 (41.2%); two cases were also tested positive for anti-HEV IgM
Positive anti-HEV IgM antibody	n = 2 (11.8%)

Liver function test (HEV seropositive cases)	anti-HEV IgG + /IgM- (n = 5)	anti-HEV IgG + /IgM + (n = 2)
AST (U/L)	143 (24–1576)	80.5 (45–116)
ALT (U/L)	124 (19–1615)	74 (32–116)
ALP (U/L)	183 (78–473)	444 (388–500)
TB (mg/dL)	0.7 (0.3–24.3)	7.95 (4.9–11)

General patient information of 17 pregnant women, including age, trimester, clinical and laboratory presentation, diagnoses, fetal complications, anti-HEV antibody test timing, and anti-HEV antibody (IgG/IgM) detection rate, were demonstrated. Continuous variables were presented in median and range. Frequencies were presented in numbers and percentages. GA (gestational age), AST (aspartate aminotransferase enzyme), ALT (alanine aminotransferase enzyme), ALP (alkaline phosphatase enzyme), TB (total bilirubin), ATV/r (atazanavir/ritonavir), HT (hypertension), UGIB (upper gastrointestinal bleeding), HELLP (hemolysis, elevated liver enzymes, and low platelet count), DFIU (dead fetus in utero), HAV (hepatitis A virus), HBV (hepatitis B virus), HEV (hepatitis E virus)

Anti-HEV IgG antibodies were detected in 41.2% (7/17), while anti-HEV IgM antibodies were detected in 11.8% (2/17) of the pregnancy group. Two patients diagnosed with acute HEV infection had hyperbilirubinemia (4.9 and 11 mg/dL) and strikingly high alkaline phosphatase enzyme (388 and 500 U/L); one of them also had elevated transaminase enzymes greater than 100 U/L (Table 7). One patient had preterm labor at gestational age (GA) 34^+5^ weeks, delivering a low birth weight infant (2,110 g). Her serum was tested positive for anti-HEV IgM antibody three days after her delivery. Another patient was tested positive for anti-HEV IgM at GA 35^+5^ weeks and gave birth to a normal birth weight infant at GA 38^+6^ weeks. Due to the limited number of patients in the pregnancy group, we could not appropriately compare liver enzyme levels and pregnancy complications among patients with different HEV serological status.

### HEV serological status in the organ transplant group

In this study, patients in the transplant group were defined as patients whose serum sample was sent from the transplant clinic, Siriraj Hospital to investigate anti-HEV antibodies. Of 1106 serum samples, 26 serum samples (2.35%) were from organ transplant group. The median age of this group was 39.5 years old (range 19–64). Of these 26 organ transplant recipients, 14 patients (53.8%) underwent kidney transplantation, eight patients (30.8%) underwent liver transplantation, and four patients (15.4%) underwent bone marrow transplantation. There were four (15.4%), four (15.4%), and 18 (69.2%) serum samples tested for anti-HEV antibodies within one month, one month to one year, and more than one year after transplantation, respectively (Table 8).

**Table 8:** Clinical and laboratory data of patients in organ transplant group during 2015–2018

Characteristics	Results
Number of organ transplant recipients	n = 26 (26 out of 1,106; 2.35%)
Age (years)	39.5 (range 19–64)
Gender (Male)	n = 17 (65.4%)
Type of organ transplantation	
Kidney	n = 14 (53.8%)
Liver	n = 8 (30.8%); due to HBV cirrhosis (n = 1), HCV cirrhosis with/without HCC (n = 3), autoimmune hepatitis (n = 1), Wilson disease (n = 2), cryptogenic cirrhosis (n = 1)
Bone marrow	n = 4 (15.4%)
Complications after transplantation	
Graft rejection	n = 6 (23.1%); kidney (n = 1), liver (n = 4), bone marrow (n = 1)
Febrile neutropenia	n = 3 (11.5%)
Clinical/laboratory presentations	
Hyperbilirubinemia	n = 6 (23.1%)
Elevation of AST or ALT > 100 U/L	n = 18 (69.2%)
Liver function test (All 26 cases)	
AST (U/L)	125 (25–915)
ALT (U/L)	243 (34–1,974)
ALP (U/L)	193 (51–677)
TB (mg/dL)	3.4 (0.3–35.4)
Timing of anti-HEV antibody test after organ transplantation	
< 1 month	n = 4 (15.4%)
1 month – 1 year	n = 4 (15.4%)
> 1 year	n = 18 (69.2%): 1–3 years (n = 8; 30.8%), 3–5 years (n = 4; 15.4%), 5–10 years (n = 6; 23.1%)
Results of anti-HEV antibody test	
Positive anti-HEV IgG antibody	n = 11 (42.3%)
Positive anti-HEV IgM antibody	n = 10 (38.5%)

Liver function test (each group)	Anti-HEV serological statuses			
	IgG-/IgM- (n = 11)	IgG + /IgM- (n = 5)	IgG-/IgM + (n = 4)	IgG + /IgM + (n = 6)
AST (U/L)	51 (28–133)	102.5 (25–474)	136 (56–915)	55 (33–117)
ALT (U/L)	119 (34–455)	156 (81–301)	251.5 (152–1974)	108 (53–386)
ALP (U/L)	178 (62–677)	150.5 (102–333)	179 (67–348)	95 (51–134)
TB (mg/dL)	1.1(0.4–35.4)	1.5 (0.5–27.6)	0.8 (0.6–2.3)	0.5 (0.3–0.7)

General patient information of 26 organ transplant recipients, including age, gender, clinical and laboratory presentation, type of organ transplantation, complications, anti-HEV antibody test timing, and anti-HEV antibody (IgG/IgM) detection rate, were demonstrated. Continuous variables were presented in median and range. Frequencies were presented in numbers and percentages. AST (aspartate aminotransferase enzyme), ALT (alanine aminotransferase enzyme), ALP (alkaline phosphatase enzyme), TB (total bilirubin), ESRD (end stage renal disease), HCC (hepatocellular carcinoma), HAV (hepatitis A virus), HBV (hepatitis B virus), HCV (hepatitis C virus), HEV (hepatitis E virus)

This study found that anti-HEV IgG antibodies were detected in 42.3% (11/26) of patients from the transplant clinic. HEV seroprevalence in bone marrow-, kidney-, and liver transplant recipients were 25% (1/4), 50% (7/14), and 37.5% (3/8), respectively. Anti-HEV IgM antibodies were detected in 38.5% (10/26) of patients from the transplant clinic. All patients with positive anti-IgM antibody (n = 10) were presented with transaminitis, in which eight patients had AST or ALT elevation greater than 100 U/L. Among this group, one was also presented with hyperbilirubinemia. Of these ten patients, eight were tested positive for anti-HEV IgM antibodies after organ transplantation for more than one year. There was no HEV infected case in bone marrow transplant recipients (0/4). The incidence of HEV infection in kidney-, and liver transplant recipients were 64.3% (9/14) and 12.5% (1/8), respectively (Table 8).

## Discussion

This retrospective laboratory-based cross-sectional study demonstrated the prevalence of anti-HEV IgG and IgM antibodies detected in the serum of patients with clinical hepatitis suspected of HEV infection at Siriraj Hospital, a tertiary hospital in Bangkok, Thailand, during 2015–2018. Anti-HEV IgG and IgM antibody detection rates were 40.82% and 11.75% in general patients (Tables 1, 2). Similar IgG and IgM antibody detection rates were found in pregnant women (Table 7); however, anti-HEV IgM antibodies were detected up to 38.5% in the organ transplant recipients (Tables 8).

Detection rates of anti-HEV IgG antibodies determine HEV seroprevalence. Recently, a nationwide seroprevalence survey of HEV in Thai blood donors published in 2019 showed a prevalence of 29.7%. [11]. The relatively high HEV seroprevalence reported in our study compared to previous studies in Thai individuals [9–11] could be due to the population recruited in the study. Our study's sera were obtained from the patients initially suspected of viral hepatitis, some of which had demonstrated transaminitis, jaundice, or had a history of chronic liver disease. A study in Argentina found a high seroprevalence of HEV in patients with cirrhosis than in healthy individuals (25% versus 4%) [17]. Therefore, our study may represent the HEV seroprevalence in Thai individuals in a hospital setting rather than the general Thai population.

Our study demonstrated the age-based difference in anti-HEV IgG positivity in 2016 and 2018, but not in 2015 and 2017. We speculated that the statistical difference was mainly affected by the low positive rate in the age group < 15 years old in the year 2016 (15.15%) and 2018 (4.35%), compared to the other 2 years (Table 3, Fig. 1B). Moreover, anti-HEV IgG positivity, depending on age, could be partly affected by the unequal amounts of samples sent to the laboratory for HEV antibody testing. Most serum samples were obtained from patients aged 40–65; relatively low sample numbers were from the age group < 15 years old, potentially creating sampling bias (Table 3, Fig. 1D).

The seroprevalence of HEV in pregnant women generally differs across geographic locations, such as 10.24–16.2% in China [18–20], 11.6—84.3% in Africa [21, 22], 3.6–29.3% in Europe [23, 24], and only 0.2% in northern Lebanon [25]. Anti-HEV IgG antibodies were detected in 41.2% in the pregnancy group in our study, which was higher than the general female patients (36.53%) (Tables 2, 7) and was relatively high compared to other geographic regions except for Africa. However, we could not exclude the effect of the small sample size (n = 17) and the nature of the study subjects recruited from a hospital setting on the seropositive detection rate in our study. Of note, IgG antibodies can remain detectable at high levels for years after infection [26]; therefore, HEV IgG seropositivity in pregnant women may result from clearance after infection before having a pregnancy status.

The seroprevalence of HEV in organ transplant recipients was estimated to range from 8.3% to 43% [8] and was geographically different. A study in kidney transplant recipients in Thailand by Payoong et al*.*, 2017 [12] demonstrated an HEV seroprevalence of 26%. Another study in France revealed an HEV seroprevalence in kidney transplant recipients presenting with clinical hepatitis of 14% [13]. However, lack of sensitivity of the commercial microplate enzyme immunoassay (MEIA) could result in underestimated HEV prevalence in their study [14]. Recently, HEV IgG seroprevalence in post-liver transplant recipients in Thailand were reported to be as high as 55.6% by Kamolmit et al. [15]. In concordance to our study, anti-HEV IgG antibodies were detected in 42.3% of the transplant group, with the highest prevalence in kidney transplant recipients (50%). Given that organ transplant recipients were accounted for 2.35% of our study population, larger sample size is required to accurately reflect the HEV seroprevalence in transplant recipients with clinical hepatitis.

The clinical manifestations of patients with HEV infection are challenging to distinguished from other types of acute viral hepatitis and hepatitis from other causes. Diagnosis of HEV infection is usually based on anti-HEV IgM detection or HEV RNA detection. Siripanyaphinyo et al., 2014 revealed a 4.4% detection rate of anti-HEV IgM antibody from patients’ sera in Bangkok during 2008–2011 [27]. Although the sample recruitment in both Siripanyaphinyo et al*.*, 2014, and our study were both from the hospital setting in Bangkok, we observed a higher incidence of HEV infection. The different sample collection timing and sample size could partly explain the higher incidence observed in our study. Besides, the ELISA test kits used for anti-HEV antibody detection in the studies (Dia.Pro in Siripanyaphinyo et al*.*, 2014 versus EUROIMMUN in our study) might have different sensitivity and specificity that could affect the detection rate as previously reported [28].

We reported the incidence of HEV infection in pregnant women of 11.8% (Table 7). Studies from China demonstrated that the anti-HEV IgM antibody's detection rate in pregnant women ranged from 0.6 to 4.11% [18–20, 29]. These studies screened pregnant women during their antenatal and postpartum visits, most of which were not presented with clinical hepatitis, unlike our study. Another study in Ghana demonstrated that the anti-HEV IgM antibody detection rate in pregnant women was 18.4% and that 76% of HEV infected cases occurred in the third trimester [30]. Like this study, we observed that both pregnant women were tested positive for anti-HEV IgM during their third trimester (GA 34–36 weeks). Therefore, using HEV serological screening in pregnant women during her third trimester in the high prevalence area could be beneficial.

The anti-HEV IgM antibody detection rate in organ transplant recipients in our study was 38.5%. Unlike our study, another study from Japan showed that anti-HEV IgM antibody detection rates in liver transplant recipients and kidney transplant recipients were 0.05% (1/1893) [31] and 0.28% (7/2526) [32], respectively. Another study in North America demonstrated that 4% (12/311) developed post-transplant HEV infection [33]. Factors that might affect the anti-HEV IgM detection rates in our study and others could be due to different sampling sizes, geographic regions, and HEV antibody test timing after transplantation. However, the serological test we used was Anti-Hepatitis E Virus (HEV) ELISA (IgM) (EUROIMMUN, Federal Republic of Germany), which only detect HEV genotypes 1 and 3. Although the main Thai isolates of HEV were previously reported as HEV genotype 3 [27], we could miss the detection of other HEV genotypes since our study did not incorporate the genotyping information. A previous study compared the sensitivity of commercial ELISA kits for antibody detection for HEV genotype 3. The sensitivity for anti-HEV IgG detection of the EUROIMMUN kit was relatively low (57.5%), while other kits, i.e., MP, Dia.Pro, WANTAI showed the sensitivity of 70%, 77.5%, and 72.5%, respectively [28]. Therefore, anti-HEV IgG antibody prevalence could be underestimated in our study. On the other hand, the sensitivity for anti-HEV IgM detection of the EUROIMMUN kit was 61.5%, comparable to other kits, i.e., MP (59.6%), Dia.Pro (59.6%), and WANTAI (65.4%) [28]. However, our study lacks HEV RNA detection information to verify acute HEV infected cases determined by anti-HEV IgM antibody, which is the limitation of our study design.

Our analysis on 796 serum samples demonstrated that ALT and total bilirubin levels, but not AST and ALP levels, showed statistically significant differences among patients with different HEV serological statuses. The results remain unchanged even when 66 serum samples, which were serologically confirmed for infection with other hepatitis viruses, were removed from the analysis [data not shown]. Patients who tested positive for anti-HEV IgM antibodies (G–M+ and G+M+) had higher ALT levels (> 300 U/L). In comparison, patients who tested positive for anti-HEV IgG (G+M−, G+M+) had more elevated total bilirubin levels (> 3.8 U/L) (Table 6).

In concordance with our study, a retrospective study of serial liver function test comparing acute HEV cases with three control groups: common bile duct stones, drug-induced liver injury, and negative HEV cases by Wallace et al*.*, 2017 showed that acute HEV cases had a significantly higher ALT than all control groups. The study suggested that patients with ALT more than 300 U/L should be investigated for HEV infection [34]. Another study in Chinese blood donors also showed the association of elevated ALT in donors with positive anti-HEV IgG antibody [35]. A study in Korea indicated that the median peak AST and ALT levels of patients with HEV infection were similar to those infected with HCV but lower than those infected with HAV and HBV. In contrast, the median peak total bilirubin levels in patients with HEV infections were similar to those infected with HAV and HBV but higher than those infected with HCV [36]. However, our study did not design to compare the liver function test among the four hepatitis viruses as the serum samples were recruited from patients initially suspected of HEV infection. Therefore, the information of other hepatitis virus serological markers in our study group was limited.

## Conclusions

Our study demonstrated the relatively high HEV seroprevalence and incidence in patients with clinical hepatitis, raising awareness for disease recognition in the Thai patients in a hospital setting. These results potentially benefit the clinicians in decision-making to investigate HEV antibodies, especially in those with elevated ALT (> 300 U/L) and facilitating proper management for patients. However, larger sample size is required to accurately reflect the HEV seroprevalence in pregnancy group and organ transplant recipients with clinical hepatitis. Further studies on the long-term effects of HEV seropositivity and HEV genotypes in patients presenting with clinical hepatitis from multi-center hospitals will provide more insight into the HEV burden in the high endemic countries.

## Data Availability

The datasets used and analyzed during the current study are available from the corresponding author on reasonable request.

## Abbreviations

ALP: Alkaline phosphatase enzyme
ALT: Alanine aminotransferase enzyme
AST: Aspartate aminotransferase enzyme
ATV/r: Atazanavir/ritonavir
DFIU: Dead fetus in utero
ELISA: Enzyme-linked immunosorbent assay
ESRD: End stage renal disease
GA: Gestational age
HAV: Hepatitis A virus
HBV: Hepatitis B virus
HCC: Hepatocellular carcinoma
HCV: Hepatitis C virus
HDV: Hepatitis D virus
HELLP: Hemolysis, elevated liver enzymes, and low platelet count
HEV: Hepatitis E virus
HT: Hypertension
TB: Total bilirubin
U/L: Units per liter
UGIB: Upper gastrointestinal bleeding
